# Radiation injury and gut microbiota-based treatment

**DOI:** 10.1093/procel/pwad044

**Published:** 2023-07-20

**Authors:** Weihong Wang, Bota Cui, Yongzhan Nie, Lijuan Sun, Faming Zhang

**Affiliations:** Department of Microbiota Medicine and Medical Center for Digestive Diseases, The Second Affiliated Hospital of Nanjing Medical University, Nanjing 210011, China; Department of Microbiotherapy, Sir Run Run Hospital, Nanjing Medical University, Nanjing 211166, China; Department of Microbiota Medicine and Medical Center for Digestive Diseases, The Second Affiliated Hospital of Nanjing Medical University, Nanjing 210011, China; Department of Microbiotherapy, Sir Run Run Hospital, Nanjing Medical University, Nanjing 211166, China; State Key Laboratory of Holistic Integrative Management of Gastrointestinal Cancers and National Clinical Research Center for Digestive Diseases, Xijing Hospital of Digestive Diseases, Fourth Military Medical University, Xi’an 710032, China; National Clinical Research Center for Digestive Diseases, Xi’an 710032, China; Key Laboratory of Resource Biology and Biotechnology in Western China, Ministry of Education, School of Medicine, Northwest University, Xi’an 710069, China; Department of Microbiota Medicine and Medical Center for Digestive Diseases, The Second Affiliated Hospital of Nanjing Medical University, Nanjing 210011, China; Department of Microbiotherapy, Sir Run Run Hospital, Nanjing Medical University, Nanjing 211166, China; National Clinical Research Center for Digestive Diseases, Xi’an 710032, China

**Keywords:** microbiome, ionizing radiation, radiation-induced injury, short-chain fatty acids, fecal microbiota transplant, washed microbiota transplantation, war, nuclear, microbiota medicine

## Abstract

The exposure to either medical sources or accidental radiation can cause varying degrees of radiation injury (RI). RI is a common disease involving multiple human body parts and organs, yet effective treatments are currently limited. Accumulating evidence suggests gut microbiota are closely associated with the development and prevention of various RI. This article summarizes 10 common types of RI and their possible mechanisms. It also highlights the changes and potential microbiota-based treatments for RI, including probiotics, metabolites, and microbiota transplantation. Additionally, a 5P-Framework is proposed to provide a comprehensive strategy for managing RI.

## Introduction

Radiation toxicities have become a worldwide concern in recent decades. The high-powered waves of radiation therapy inevitably harm healthy cells and thus cause some radiation toxicities (Kordahi and Chassaing 2021). Additionally, the Chernobyl and Fukushima nuclear power plant leaks, global nuclear war, and terrorist attacks related to nuclear weapons have exposed humanity to unprecedented levels of radiation, resulting in a range of health threats, such as mental health issues, anxiety, and increased morbidity (Berwick and Shine 2020, Hande et al*.* 2023). Therefore, the exposure to radiation, whether through medical treatment or accidental means (e.g., industrial accidents and nuclear catastrophes), can have detrimental effects on human health.

Generally, radiation toxicities are regarded as radiation injury (RI) or radiation-induced injury. RI can induce both systemic effects (e.g., fatigue and emaciation) and gastrointestinal symptoms (e.g., vomiting, diarrhea, and hematochezia). The true incidence of RI is challenging due to a lack of consensus on diagnostic criteria and patient feedback. However, it is estimated that up to 90% of patients experience gastrointestinal symptoms or discomfort in the first few weeks after receiving radiation to their abdominopelvic region (Hauer-Jensen et al*.* 2014).

Gut microbiota, as well as microbe-derived metabolites represented by short-chain fatty acids (SCFAs), regulate host metabolism and immunity as well as maintain the homeostasis and internal environment stability (Guo et al*.* 2020). In recent years, extensive preclinical and clinical studies have highlighted that gut microbes and their metabolites play a significant role in cancer and oncotherapy-related diseases, which has sparked great interest among researchers (Routy et al*.* 2018, Wang et al*.* 2018, Baruch et al*.* 2021, Davar et al*.* 2021). Among them, radiation therapy can significantly alter the gut microbiome, leading to an imbalanced and less diverse microbiota community. This dysbiosis has been linked to increased inflammation, oxidative stress, and tissue damage. It is worth noting that RI is a more significant clinical issue than previously acknowledged, with radiation-induced intestinal injury being pathologically similar to inflammatory bowel disease (IBD), but with a high prevalence. Given this, exploring effective treatments for RI is crucial, and the role of gut microbiota shows great potential in this regard. This review provides insights into the prevention of microbiota-based treatments for RI, and enriches the scientific explantations of microbiota medicine of a branch discipline (Zhang et al., 2023).

## Radiation toxicity and the role of microbiota

**Figure 1.**
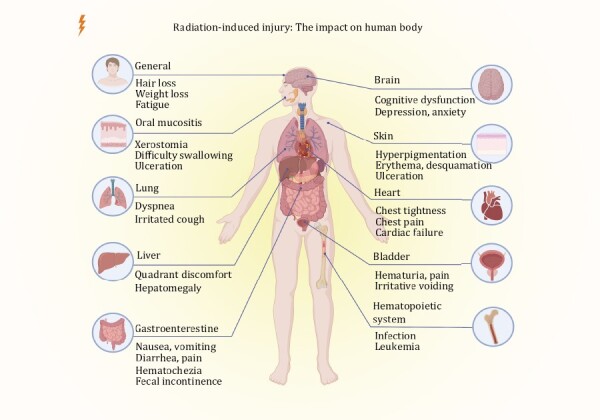
The impact of radiation-induced injury on the human body. Common symptoms caused by radiation damage to different tissues or organs are listed.

Radiation disrupts the intestinal microbiota composition and promotes dysbiosis, which may contribute to radiation toxicity (Al-Qadami et al*.* 2019). The different damages on the human body with radiation have shown in Fig. 1. It is important to note that some systemic symptoms, like fatigue, weight loss, and cancer-induced malnutrition, may occur before radiation and therefore are not highlighted in this discussion.

### Radiation-induced skin injury

The skin is the largest organ of the human body and performs various critical biological functions. Due to its high rate of dividing cells, the cutaneous epithelium is extremely susceptible to RI (Ashack et al*.* 2020). Acute radiation skin injury includes erythema, hyperpigmentation, dry desquamation, alopecia, moist desquamation, and ulceration. The incidence of chronic adverse effects, such as delayed ulcers, fibrosis, ischemia, atrophy, and cutaneous malignant changes, tend to be underestimated (Soriano et al*.* 2019).

A clinical study conducted by Ramadan et al*.* (2021) specifically focused on radiotherapy-induced dermatitis and observed that the overrepresentation of *Pseudomonas*, *Staphylococcus*, and *Stenotrophomonas* was markedly associated with delayed healing of radiation-induced skin injury. Furthermore, Janko et al*.* (2012) found that mice deficient IL-1 or the IL-1 receptor exhibited reduced inflammation and suffered decreased severity of radiation dermatitis. Given the positive role of probiotics in the prevention of skin diseases such as atopic dermatitis, microbial-mediated treatment may be a viable option for radiation-induced skin injury (Kim et al*.* 2021, Trompette et al*.* 2022).

### Radiation-induced brain injury

Radiation-induced brain injury is a common complication of brain irradiation, which may cause cognitive dysfunction and neuroinflammation. Anxious-depression-like behaviors are among the side effects of brain radiotherapy (Xu et al*.* 2022). Research has shown the involvement of gut microbes in brain function and their contribution to alter behavior, mood, and the pathogenesis of certain neurological conditions in view of the gut–brain axis. Gut microbial alteration induced behavioral impairment by decreasing adult neurogenesis and long-term potentiation of synaptic transmission, and altering the gene expression profile in the hippocampus (Liu et al*.* 2022a). In addition, gut microbiota or metabolites through supplementation or suppression may have neuroprotective effects against radiation-induced brain injury. A 4-week supplementation of Eleutheroside E was administered to irradiated mice, resulting in significant alterations in the relative abundance of *Lactobacillus* and *Helicobacter*. This modulation was achieved through the activation of the PKA/CREB/BDNF signaling pathway. Importantly, Eleutheroside E supplementation improved the cognition and spatial memory impairments along with protecting hippocampal neurons (Song et al*.* 2022).

### Radiation-induced oral mucositis

The salivary glands, particularly the parotid glands, are frequently irradiated during the treatment of head and neck tumors. The parotid glands contain serous cells that are radiosensitive and undergo apoptosis. Radiation-induced oral mucositis (ROM) is a common side effect caused by radiotherapy of head and neck cancers, with more than 90% of patients who receive radiation doses between 50 Gy and 54 Gy suffering from ROM (Elting et al*.* 2007). Xerostomia, difficulty swallowing and oral ulceration can be observed in a great number of severe patients with ROM (van der Laan et al*.* 2021).

Radiotherapy can cause distinct shifts in the oral microbiota. Zhu et al. found that patients in the severe group had a significantly higher abundance of *Streptococcus* before erythema became visible. They also found that from the onset of visible erythema to the beginning of severe mucositis, severe patients had significantly less diverse microbiota and a higher abundance of *Actinobacillus* than mild patients (Zhu et al*.* 2017). Recently, accumulated evidence has revealed that oral and intestinal microbiomes exert a great influence on the development of ROM. Al-Qadami et al*.* (2022) found that the percentage of the mucosal ulcer in rats treated with 20 Gy X-ray radiation and antibiotic-induced microbiota depletion were lower than the radiation alone group, which revealed that the gut microbiota plays a role in ROM pathogenesis. In addition, oral microbiota transplantation (OMT) can mitigate radiation-induced oral mucositis (Xiao et al*.* 2021). However, the other study from Cui’s team found OMT in colorectal cancer impaired the therapeutic efficacy of radiotherapy and disrupted the composition of gut microbiota (Dong et al*.* 2021). Therefore, the feasibility and safety of OMT needs to be demonstrated in numerous clinical trials as the application of OMT is still in its early stages.

### Radiation-induced lung injury

Radiation-induced lung injury (RLI) is one of the most common, severe, and intractable toxicities of thoracic radiotherapy, characterized by damage to alveolar cells and an over-reaction inflammation (Weng et al*.* 2019). When RLI and pulmonary fibrosis appear in the early stage, dyspnea can be observed clinically, but vascular injury and atrophy will occur in the late stage.

After radiation therapy, mice with gut microbiota disequilibrium tend to exhibit more pathological severe lung damage compared to those without antibiotic treatment. Following irradiation, there was an observed increase in the levels of *Alisipes*, *Mucispirillum*, *Helicobacter*, *Turibacter*, *Parabacteroides*, *Lachnoclostridium*, and *Intestinimonas* in the intestinal microbiota. Conversely, the levels of *Alloprevotella*, *Muribaculum*, *Anaerotruncus*, *Enterococcus*, *Bacteroides*, *Ruminiclostridium*, *Lactococcus*, and *Lactobacillus* decreased (Chen et al*.* 2021a). Schuijt et al. observed that after fecal microbiota transplantation (FMT), the intestinal microbiota played a protective mediator in pneumococcal pneumonia by reducing the level of TNF-α and IL-10. These shed light on the interaction between the intestinal microbiota and pneumonia (Schuijt et al*.* 2016). A recent study demonstrated that FMT in irradiated mice led to an improvement in radiation-induced inflammation (Nie et al*.* 2020). In addition, Imidazole propionate (ImP, a secondary metabolite of gut microbiota-derived l-Histidine), and gut microbiota-derived PGF2α were found to promote lung cell growth in irradiated mice following FMT (Chen et al*.* 2021a, b). Together, these findings pave the way for the clinical treatment of RLI.

### Radiation-induced heart injury

The heart inevitably receives some radiation in undergoing thoracic radiotherapy due to the anatomical proximity, which results in acute and chronic cardiotoxicity, and ultimately leads to heart failure, coronary artery disease, pericardial, and valvular heart disease. Of note, any dose of radiation is unsafe for cardiovascular structures. The risk of heart injury increases by 7.4% when the radiation dose increases by 1 Gy (Darby et al*.* 2013).

Gut microbiota and their metabolites have played a positive role in the protection of heart disease in previous studies. In two different mouse models of hypertensive cardiovascular damage, propionate was shown to significantly attenuate cardiac hypertrophy, fibrosis, and vascular dysfunction (Bartolomaeus et al*.* 2019). As mentioned before, FMT can increase Imp, thus improving heart systolic function, retarded the pathological process of heart tissues, as well as combat the reduction of gut microbiota (e.g., *Akkermansia_muciniphila*, *Lactobacillus_reuteri*, *Clostridium_sp_Cuiture-41*, *Lachnospiraceae_bacrerium_615*, *Ileibacterium_valens*, and *Helicobacter_bills*) after total chest radiation through inhibiting cell pyroptosis (Chen et al*.* 2021a).

Additionally, intestinal microbiota has made much progress in cardiotoxicity caused by chemotherapy. Phenylalanine-butyramide and FMT protect the heart from Dox cardiotoxicity by decreasing oxidative stress and improving mitochondrial function (Russo et al*.* 2019, An et al*.* 2021). Hence, these evidence from preclinical studies provide a prospective direction for practical methods.

### Radiation-induced gastrointestinal injury

The gastrointestinal tract is an extremely radiosensitive organ. As a part of the standard course of treatment for ovarian, prostate, colon, gastric, and bladder cancers, radiotherapy caused the most common clinical adverse effect, radiation-induced gastric injury, duodenitis, enteritis (RE), or proctitis. From a long-term perspective, the majority of patients have persistent or recurrent symptoms, and about 10% die directly from radiation enteritis (RE) (Hauer-Jensen et al*.* 2014). Typical symptoms of radiation-induced gastrointestinal injury include nausea, vomiting, diarrhea, abdominal pain, hematochezia, flatulence, and fecal incontinence.

Evidence supporting the pivotal role of the gut microbiota in the development of radiation-induced gastrointestinal injury is growing. With analysis of fecal samples from 18 cervical cancer patients during radiotherapy, Wang et al. indicated that the relative abundance of *Proteobacteria* and *Gammaproteobacteria* increased, but *Bacteroides* reduced. Moreover, the more abundant *Virgibacillus* and *Alcanivorax* are also observed in patients with mild RE (Wang et al*.* 2019). Similarly, a large pilot study significantly revealed higher counts of *Clostridium IV*, *Roseburia*, and *Phascolarctobacterium* in pelvic patients with RE (*n* = 134) (Reis Ferreira et al*.* 2019). Liu et al*.* (2021) analyzed the fecal samples from RE patients (*n* = 32) with chronic radiation proctitis. Compared with non-hematochezia patients, the relative abundance of *Peptostreptococcaceae*, *Eubacterium*, and *Allisonella* were increased significantly. The result implies these bacteria may be a potential risk for RE patients caused hematochezia. Based on the link between gut microbiota and RE, Riehl et al*.* (2019) reported that *Lactobacillus rhamnosus* GG (LGG) could prevent the occurrence of diarrhea in patients receiving radiotherapy. As a metabolite of the intestinal microbiota, Urolithin A (UroA) shows immunomodulatory and anti-inflammatory capacity in RE (Zhang et al*.* 2021).

### Radiation-induced liver injury

Although hepatectomy is currently considered as the most effective therapy for hepatocellular carcinoma (HCC), radical curative surgery is ineligible for some patients with advanced cancer (Karaman et al*.* 2014). Radiotherapy is recommended as a locoregional treatment option for inoperable HCC in international guidelines. However, radio-sensitivity of the liver has become a major limitation of radiotherapy in the treatment of HCC or other abdominal tumors. Moreover, radiation-induced liver damage (RLD) has a significant mortality rate (Koay et al*.* 2018). Classic RLD, characterized by occasional right upper quadrant discomfort anicteric ascites and hepatomegaly, is unlikely to occur in patients with baseline Child-Pugh A liver function if treated with doses of ≤30 Gy in 2 Gy per fraction. On the other hand, non-classic RLD is a spectrum of liver toxicity, including a general decline in liver function and elevation of liver enzymes. It is hard to identify and predict, especially in patients with underlying liver disease (Munoz-Schuffenegger et al*.* 2017).

Miousse et al. found that mice with radiation fed a methionine-supplemented diet are easy to suffer acute RLD. Analysis of the intestinal microbiome demonstrated the high abundance of *Burkhoderiales* in the gut microbiota taxa, along with the development of the leaky gut syndrome and bacterial translocation into the liver. The probable mechanism involves the dominant role of downregulating tight-junction-related proteins, and influences the one-carbon metabolism pathway and amino acid levels (Miousse et al*.* 2020).

### Radiation-induced cystitis

Radiation-induced cystitis (RC) is a late-onset and under-reported condition after pelvic radiotherapy with an incidence rate of 5%–10%, even higher (David et al*.* 2022). RC tends to be clinically severe and can cause extreme pain, hematuria, and irritative voiding symptoms. An animal study by Oscarsson et al*.* (2017) indicated that hyperbaric oxygen therapy (HBOT) might prevent radiation-induced changes by affecting oxidative stress and radiation-induced inflammatory cascades.

Limited number of studies on the association between intestinal microbiota and RC. Some specific bacterial communities (such as *Lactobacillus* spp., *Proteus mirabilis*) exist in the healthy urinary tract, and changes in these microbes have been observed in certain urologic disorders such as urinary tract infections, urologic cancers, and chronic prostatitis, etc. (Aragón et al*.* 2018, Szczerbiec et al*.* 2022).

### Radiation-induced hematopoietic system injury

Bone marrow is a tissue with relative immaturity, high metabolic activity and mitotic activity, so it is susceptible to radiation, which leads to myelodysplasia and hematopoietic system injury (Green and Rubin 2014). Leukemia can occur in some people under both high- and low-dose irradiation. Considering that the prevalence and severity of leukopenia in cervical cancer patients receiving radiotherapy were underrated, and those with low baseline leukocyte count are more likely for leukopenia, early prevention may be needed during radiation.

Lucas et al*.* (2018) have shown that microbiota-derived SCFAs can regulate osteoclast differentiation through metabolic reprogramming thereby stimulating hematopoietic cell regeneration. Besides, propionate rendered mice resistant to radiation by attenuating DNA damage and releasing reactive oxygen species (ROS) in hematopoietic and gastrointestinal tissues (Guo et al*.* 2020). Oral administration of lactic acid-producing bacteria activated stem cell factor secretion from leptin receptor-expressing (LepR^+^) in bone marrow mesenchymal stromal cells and subsequently accelerated hematopoiesis and erythropoiesis (Lee et al*.* 2021). Moreover, FMT from young mice rejuvenated aged hematopoietic stem cells (HSCs) with enhanced short-term and long-term hematopoietic repopulation capacity. Mechanistically, FMT activated the FoxO pathway, and promoted lymphoid differentiation in aged long-term HSCs. Additionally, tryptophan-associated metabolites were significantly upregulated after FMT in aged mice (Zeng et al*.* 2023). These studies revealed the role of gut microbiota in hematopoiesis using animal models.

## Mechanisms linking radiation and microbiota

**Figure 2.**
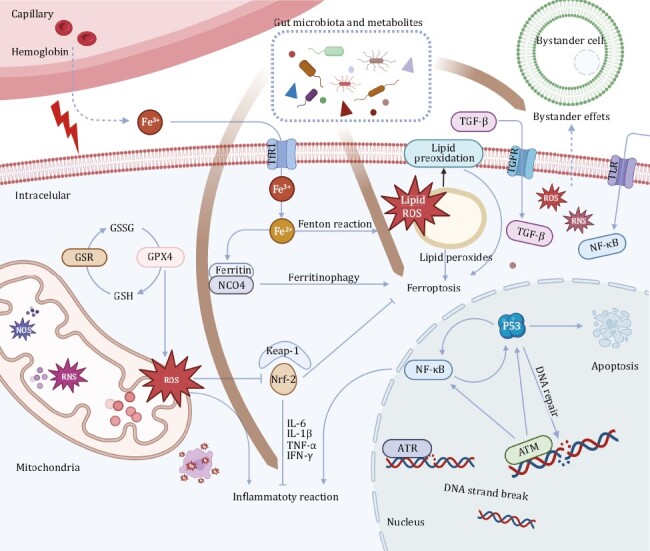
Gut microbiota are involved in the potential mechanisms underlying RI at the molecular and cellular levels. Inflammatory reaction caused by DNA damage and reactive oxygen species generation, ferroptosis caused by lipid peroxidation, and radiation-induced bystander effects is the possible mechanisms lead to RI.

Direct and indirect effects of radiation, when combined, initiate a cascade of biochemical and molecular signaling activities, leading to damage or irreversible physiological changes in the cell or its death. The pathogenesis of this damage is multifactorial and interacts with each other, and is far more complex than previously assumed. Despite the lack of a full understanding of the specific mechanism involved, there are several proposed mechanisms at molecular and cellular levels. DNA damage, ROS generation, lipid peroxidation, and radiation-induced bystander effects (RIBE), lead to the activation of pro-inflammatory signaling pathways and the aggregation of inflammatory cells (Fig. 2).

### Double-strand breaks of DNA and NF-κB pathway actives

DNA is the most critical target for cell survival as it carries the genetic information necessary for the functioning and reproduction of cells. When cells are exposed to radiation, the energy carried by radiation emissions can directly disrupt DNA structures, leading to single and double-strand breaks (DSB). This triggers the activation of multiple protein kinases and signaling pathways. In response to DNA strand breaks caused by ionizing radiation, the ataxia telangiectasia mutated (ATM) protein is activated by chromatin structural changes (Morgan and Kastan 1997), and then activates the p53 tumor suppressor protein in the cell nucleus, which in turn activates the NF-κB pathway (Jonak et al*.* 2016). The activation of the NF-κB pathway will release a series of pro-inflammatory cytokines to promote inflammation.

Gut microbiota and SCFAs may mitigate RI by modulating p53 and NK-κB pathways. Butyrate has been reported exerts anti-inflammatory effects by inhibiting the NF-κB pathway (Liu et al*.* 2019). Isorhamnetin, a flavonoid and a subclass of polyphenols, has been found to moderate p53 activity and promote the phosphorylation of ATM, leading to enhanced 53BP1 (the mediator protein for repairing DSB) recruitment in irradiated cells, and improve survival in mice subjected to a lethal dose of abdominal irradiation (Shibata and Jeggo 2020). However, the radioprotective effect of isorhamnetin was not observed in the presence of an ATM inhibitor, indicating its protective effect is dependent on ATM (Nishiyama et al*.* 2021). UroA, a metabolite of the intestinal microbiota of ellagitannin, is associated with the recovery of the radiation-induced intestinal microbiota profile changes in mice. UroA has been found to inhibit p53-mediated apoptosis and remodel the gut microbes (Zhang et al*.* 2021). In addition, Abdelazeem et al. found that UroA inhibits IκBα phosphorylation and suppresses MAPK and PI3K activation by suppressing the NF-κB pathway. In this case, it also preserves DNA by maintaining intracellular calcium and ROS homeostasis (Abdelazeem et al*.* 2021).

### Oxidative stress and inflammatory effects

Water is the majority component of cells and is the most likely target of radiolysis by high-energy photons, aside from DNA. Ionizing radiation causes the radiolysis of water and stimulates nitrogen oxide synthetase to produce ROS and reactive nitrogen species (RNS) in the mitochondrion, respectively (Turrens 2003). Excessive ROS/RNS production is a harmful process that may trigger chemical chain reactions with all major cellular macromolecules such as DNA, proteins, and membrane lipids (Spyropoulos et al*.* 2011). Nuclear factor erythroid 2-related factor 2 (Nrf-2) and Kelch-like ECH-associated protein 1 (Keap1) belong to the endogenous antioxidant pathway, which can alleviate the excessive accumulation of ROS. However, Nrf-2 deficiency aggravates radiation-induced histopathological changes, macrophage and neutrophil infiltration, serum levels of pro-inflammatory cytokines (IL-6, IFN-γ, TNF-α, and IL-1β), and the levels of peroxidation products (Tian et al*.* 2018).

Butyrate and other gut microbiota-derived metabolites, such as PGF2α and phenylalanine-butyramide, have been shown to alleviate toxicity by reducing oxidative stress and ameliorating mitochondrial function (Russo et al*.* 2019). Additionally, the absence of anti-inflammatory microbiota (e.g., *Bifidobacterium* and *Faecalibacterium prausnitzii*) in patients undergoing radiation or chemotherapy may contribute to the onset of inflammatory events (Tian et al*.* 2020).

### Lipid damage and ferroptosis

While DNA is the primary target of radiation, significant damage to other cellular molecules, such as proteins and lipids, is also produced simultaneously. Ferroptosis is a type of regulated cell death induced by lipid peroxidation, characterized by the depletion of glutathione and the decrease of glutathione peroxidase-4 (GPX4) activity. Specifically, lipid oxides cannot be metabolized through the glutathione reductase reaction catalyzed by GPX4, leading to the oxidation of lipids by divalent iron ions, producing ROS and the onset of ferroptosis (Melin et al*.* 2022). Recent evidence has shown that radiation is capable of inducing ferroptosis in intestinal epithelial cells, and suggested the complex interplay between ferroptosis, ionizing radiation, ATM, and p53 (Su et al*.* 2022). Zhang et al*.* (2022) suggested that the radiation-induced intestinal injury is associated with the activation of the NF-κB pathway by ferroptosis. Additionally, activation of the Nrf-2 pathway has been shown to blunt ferroptosis and thus acts as a protective factor (Li et al*.* 2022).

Deng et al*.* (2021) have found that a kind of gut microbiota metabolite, capsiate, can enhance GPX4 expression to protect from the ferroptosis-dependent intestinal ischemia/reperfusion injury. These shreds of evidence provide evidence of a potential relationship between gut microbiota and ferroptosis.

### Radiation-induced bystander effects

Cells exposed to radiation and other genotoxic agents (targeted cells) can communicate their DNA damage response status to cells that have not been directly irradiated (bystander cells). These targeted cells can induce DNA damage in non-targeted bystander cells, thereby threatening their genomic stability and increasing the risk of cancer. This phenomenon, known as radiation-induced bystander effects (RIBE), has gained great attention recently (Klammer et al*.* 2015).

RIBE activates multiple signaling pathways in bystander cells, including NF-κB, MAPK, and JNK, leading to altered expression of stress response genes, activation of DNA damage repair, proliferation, apoptosis, and death of bystander cells (Wang et al*.* 2021). Hu et al. have revealed acute negative bystander effects of irradiated recipients on transplanted mouse HSCs (Shen et al*.* 2012). Their further analyses showed that the RIBE-affected human hematopoietic cells exhibited enhanced DNA damage responses, cell-cycle arrest, and p53-dependent apoptosis, primarily due to oxidative stress (Hu et al*.* 2021).

## Gut microbiota-based treatment for radiation injury

### Changes in gut microbiota after radiation

**Figure 3.**
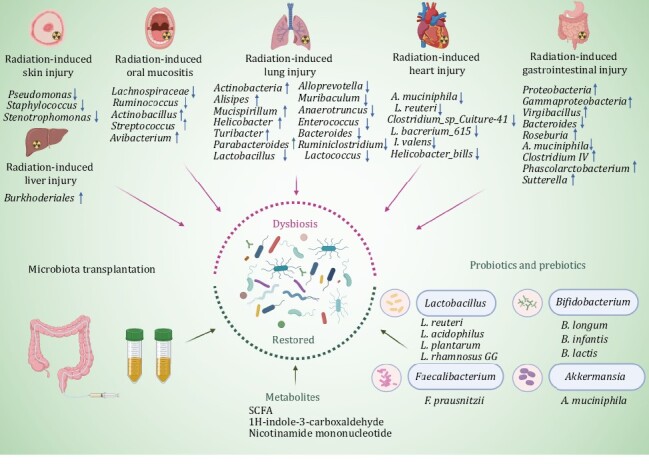
The development and treatment of RI can be regulated by gut microbiota. Different RI demonstrated obvious dysbiosis of gut microbiota. RI can be regulated by probiotics, prebiotics, and microbiota transplantation.

Generally, mice can hardly survive for a long time after high-dose radiation. However, a study by Ting’s group observed that a small part of mice could be free from a high dose of radiation and live an average life span, and the key reason is that these mice developed a distinct gut microbiome after radiation (Guo et al*.* 2020). Among them, *Lachnospiraceae* and *Enterococcaceae* are the most enriched taxa in the fecal samples of survivors. They are associated with post-radiation restoration of hematopoiesis and gastrointestinal repair. In addition, two tryptophan pathway metabolites,1H-indole-3-carboxaldehyde and kynurenic acid, provided long-term radioprotection *in vivo*. Gerassy-Vainberg further observed that the expression of some inflammatory cytokines (TNF-α, IF-1β, and IL-6) increased in germ-free mice models that underwent FMT from irradiated fecal microbiota (Gerassy-Vainberg et al*.* 2018). As shown in Fig. 3, the above evidence from clinical and animal studies indicated the association between gut microbiota and radiation-induced injury. Therefore, gut microbiota is the future direction for treating RI.

### Animal and clinical findings from microbiota transplantation

Fecal microbiota transplantation, an efficient way of restoring and reconstructing the composition and function of gut microbiota, is the crucial motivation of research related to the interaction between microbiota and diseases. FMT has been reported as an effective treatment for dysbiosis-associated diseases, such as *Clostridioides difficile* (*C*. *difficile*) infection, IBD, refractory diarrhea, and other disease or related complication beyond the intestine (Zhang et al*.* 2018). A systematic review analyzed the effectiveness of all clinical FMT uses in 85 specific conditions worldwide from 2011 to 2021 (Wang et al*.* 2022). However, the safety of FMT is still a concern for broad clinical application. A recent systematic review reported that the rate of FMT-related adverse events (AEs) was 19% from 2000 to 2020 (Marcella et al*.* 2021). Although the majority of AEs were mild and self-limiting, FMT should be improved to reduce the AEs. The improved methodology of FMT termed as “washed microbiota transplantation” (WMT) was released by the FMT-standardization Study Group consensus statement in 2019 (Zhang et al*.* 2018). Based on an automatic filtration and washing process and the related delivery, WMT could wash out more viruses and pro-inflammatory mediators to improve the safety of FMT (Zhang et al*.* 2020, Lu et al*.* 2022). WMT is the new generation of FMT, which decreased the incidence of AEs without reducing the clinical efficacy compared to manual FMT (Zhang et al*.* 2020). WMT can be delivered through upper-gut, mid-gut and lower gut, including capsule, gastroscopy, mid-gut tube, colonoscopy, colonic transendoscopic enteral tube, and enema (Wang et al*.* 2023b).

Washed preparation for WMT is processed using the device approved by China. Notably, the fecal microbiota product named RBX2660 has been approved by FDA as the live biotherapeutic drug for the treatment of recurrent *C*. *difficile* infections via enema (Khanna et al*.* 2022). Additionally, SER-109 has also received FDA approval as microbiota-based oral therapeutic for the prevention of recurrent *C*. *difficile* infections (Cohen et al*.* 2022, Feuerstadt et al*.* 2022). The available evidence supports the feasibility of gut microbiota-based treatments for the management of RI in the future.

#### Animal model

FMT played a potential role in alleviating various RI. Nie et al*.* (2020) found that the inflammation and radiation-induced pneumonitis in irradiated mice were improved obviously and the increasing abundance of diversity, composition and community structure of gut microbiota in mice were observed after FMT. Likewise, in animal studies conducted by Cui’s group, it was demonstrated that FMT potential in mitigating radiation-induced toxicities in lung, heart, and hematopoietic system. The research findings revealed that FMT could increase the survival rate of mice following lethal dose radiation, improve gastrointestinal tract function and intestinal epithelial integrity in irradiated mice (Cui et al*.* 2017, Xiao et al*.* 2020). In total, FMT demonstrated the ability to improve inflammation and alleviate toxicity in irradiated mice, thereby mitigating RI.

#### Clinical evidence

Ding *et al.* first proved that three of five patients achieved clinical response after WMT, and found that the intestinal microbiota diversity of all radiotherapy patients increased after WMT treatment. Radiation-induced rectum edema was obviously alleviated after 8 weeks of WMT, and the beneficial bacteria such as genus *Alistipes*, *Phascolarctobacterium*, *Streptococcus*, and *Bacteromides* expanded, whereas the abundance of *Faecalibacterium* decreased (Ding et al*.* 2020). In addition, WMT brought some additional benefits to patients, such as improving the symptoms of hepatic encephalopathy and offering good intestinal conditions for the following fistula surgery. A case reported that all symptoms of chronic hemorrhagic radiation proctitis in a female patient (e.g., hematochezia, abdominal pain, and diarrhea) are obviously relieved after four courses of FMT (Zheng et al*.* 2020). Although the clinical experience and data are still limited, these studies may provide supporting evidence for the protective effects of FMT in RI.

### Animal and clinical findings from probiotics treatment

Accumulating evidence suggests that probiotics and prebiotics could promote the diversity and increase the species of gut microbiota, therefore ensuring the integrity of the intestinal mucosal barrier to avoid bacterial translocation (Reiff and Kelly 2010). A narrative literature review, which identified 60 clinical studies examining various nutritional compounds and 20 examining probiotics, pointed out that probiotics can reduce the burden of intestinal mucositis and treatment-induced diarrhea (Thomsen and Vitetta 2018). Some SCFA-producing probiotics, especially *Lactobacillus*, *Bifidobacterium*, *Faecalibacterium prausnitzii* (*F*. *prausnitzii*), and *Akkermansia muciniphila* (*A*. *muciniphila*), are reported to affect RI positively.

#### Lactobacillus

*Lactobacillus* spp. and its components modulate immune responses mainly through the exchange of immunological signals between the gastrointestinal tract and distant organs. Ki et al*.* (2014) found that *Lactiobacillus acidophilus* (*L*. *acidophilus*) is effective in shortening small intestinal mucosa damaged by limited radiation (≤15 Gy), but ineffective in more than 20 Gy. In addition, *Lactobacillus plantarum* (*L*. *plantarum*) can alleviate irradiation-induced intestinal injury by activating FXR-FGF15 signaling in intestinal epithelia to prevent mice from radiation-induced death (Jian et al*.* 2022). *Lactobacillus reuteri*, known as a second-generation probiotic, can stabilize the number and capacity of Lgr^+^5 intestinal crypt stem cells and protect intestinal microvascular endothelial cells from death by producing metabolites and releasing IL-22, thus directly inhibiting the growth of intestinal pathogens (Espinal et al*.* 2022, Hamade et al*.* 2022). *Lactiobacillus acidophilus* could enhance intestinal epithelial function with respect to irradiation-induced intestinal damage by improving intestinal stem cell function and cell differentiation (Sittipo et al*.* 2020). LGG can release radioprotective LTA (a TLR2 agonist), which protects epithelial stem cells by triggering a multicellular, adaptive immune signaling cascade involving macrophages and PGE2 secreting MSCs (Riehl et al*.* 2019).

#### Akkermansia muciniphila

*Akkermansia muciniphila* as a kind of next-generation probiotics in metabolic diseases and tumor immunotherapy has been recognized widely. It could ameliorate colitis by upregulating RORγt^+^ Treg cell-mediated immune responses, and also blunt colitis-associated colorectal cancer by reducing CD8^+^ cytotoxic T lymphocytes, TNF-α and programmed death 1 (PD-1) (Wang et al*.* 2020, Liu et al*.* 2022b). *Akkermansia muciniphila* administrated to irradiated mice could mitigate intestinal toxicity significantly, and settle in the digestive tract of mice with more serious intestinal toxicity stably (Wang et al*.* 2023a). Although the positive role of *A*. *muciniphila* and its metabolites in intestinal inflammation has been discovered, *A*. *muciniphila* may be related to the occurrence and development of intestinal diseases according to studies in mice with specific gut microbiota, certain genotype, and colorectal cancer, or in animal models infected with a specific pathogen. Kim et al*.* (2015) showed that the relative abundance of *A*. *muciniphila* in mice was elevated obviously after radiation therapy. Radiation therapy may induce enteritis symptoms, which promotes *A*. *muciniphila* to secret mucus. In addition, tumor-bearing mice show enrichment in operational taxonomic units affiliated with members of *A*. *muciniphila* (Zackular et al*.* 2013). Dingemanse et al*.* (2015) found that colonization of pathogen-low FabplCre; Apc^15lox/+^ mice with *A*. *muciniphila* increased the number of intestinal tumors. These results revealed that *A*. *muciniphila* and its related components might exacerbate pathogenic infections and inflammation of the intestine in some specific cases, although it is beneficial to the maintenance of intestinal homeostasis of the host in normal.

#### Faecalibacterium prausnitzii

*Faecalibacterium prausnitzii*, is regarded as a kind of anti-inflammatory bacterium and plays an important effect in the treatment of IBD and colitis. It has been reported that the abundance of *F*. *prausnitzii* is decreased in patients with IBD compared with healthy donors. Specifically, Zhou et al*.* (2018) found that the supernatant of *F*. *prausnitzii* regulated T helper 17 cell/regulatory T cell differentiation, and further revealed butyrate (the production of *F*. *prausnitzii*) exerts significant anti-inflammatory effects in rat models. Lapiere et al*.* (2020) observed that in rats with 29 Gy local irradiation before and after 3 days, the oral administration of *F*. *prausnitzii A2–165* strain reduced the severity of the morphological change of crypts, preserved the pool of stem/progenitor cells and the proliferating epithelial crypt cells, as well as increased production of IL-18 by colonic crypt epithelial cells.

#### Prebiotics or probiotics combinations

With the benefit of single probiotic have been reported largely, the combination of probiotics has been used in humans and animals to explore their effects on RI. The probiotic VSL# 3 is a mixture of eight probiotics, which has been used safely and broadly for gastrointestinal disease. A double-blind, placebo-controlled trial performed by Delia et al*.* (2007) verified that fewer VSL#3 patients suffered radiation-induced diarrhea (77/243, 31.6% vs. 124/239, 51.8%; *P* < 0.001) and grade 3 or 4 diarrhea compared with the placebo group (1.4% vs. 55.4%; *P* < 0.001). Dixentil, a mixture of prebiotics and probiotics (Galacto-oligosaccharides, *L*. *acidophilus*, and *Lactobacillus casei*), showed positive effects in protecting the patients with radiation-induced diarrhea (Scartoni et al*.* 2015). Compound probiotics, including *L*. *acidophilus*, *Lactobacillus rhamnosus*, *L*. *plantarum*, *Bifidobacterium longum*, and *Bifidobacterium lactis*, were found to play a positive role in acute radiation-induced intestinal injury in mice model (Zhao et al*.* 2021). *Lactobacillus acidophilus* plus *Bifidobacterium longum* can decrease grade 2–3 diarrhea and improve stool consistency caused by RE, especially in patients with pelvic cancers after external beam pelvic radiotherapy. Additionally, anti-diarrheal medication use was significantly reduced in the probiotic group (*P* < 0.05) (Chitapanarux et al*.* 2010, Linn et al*.* 2019).

#### Other prebiotics and probiotics

Wang et al*.* (2023c) showed that combing IVIg with *Trichospirillaceae* can significantly alleviate radiation-induced hematopoietic system injury and RE in male mice. Intestinal microbiota-produced valeric acid oral administration could protect hematogenic organs, and improve gastrointestinal tract function and intestinal epithelial integrity in irradiated mice (Li et al*.* 2020). Additionally, microbiota-derived l-Histidine and Imp were found to treat the radiation-induced cardiopulmonary injury (Chen et al*.* 2021a).

## The 5P-Framework: personal protection, public education, precise technology, policy making, partnership

**Figure 4.**
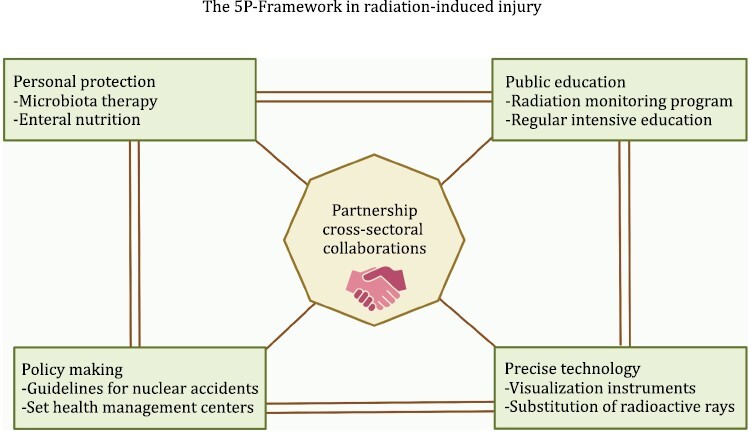
The general diagram of 5P-Framework in radiation-induced injury. The 5P-Framework includes personal, public, precise technology, policy, and the partnership which is the bridge that links various sectors.

In addition to the population requiring radiation therapy, sudden nuclear war or nuclear disaster and living or working in highly radioactive environments (hospitals, nuclear power industry, space) may damage human health. Most of these effects are a chronic process (Shuryak et al*.* 2017). Blood analysis of post-atomic bomb survivors found that the level of the inflammatory markers C-reactive protein, ROS, and IL-6 were significantly increased, and the risk of cardiovascular disease and other non-caner diseases increased. The radiation exposure, in conjunction with natural aging, may enhance the persistent inflammatory status of A-bomb survivors (Hayashi et al*.* 2012). Note that a considerable increase in leukemia risks was the most striking late effect of radiation exposure (Hsu et al*.* 2013). Moreover, in a long-term cohort study, 22,538 first primary solid cancer cases were identified, 992 of which were associated with radiation exposure. It also showed that the risk of solid cancers remained elevated more than 60 years after radiation exposure (Grant et al*.* 2017). Therefore, reducing or even avoiding the adverse effects of radiation is a widespread concern worldwide. The 5P-Framework is proposed to emphasize the protection and prevention of RI, calling for the participation of the whole society (Fig. 4).

### Personal protection

An important challenge of modern radiation therapy is to develop pharmaceutical interventions capable of modifying the normal response of a biological system to ionizing radiation. Some microbiota can be applied in RI as a mainstream treatment (Spyropoulos et al*.* 2011). Diverse bacteria inhabiting extremely radioactive waste and disaster sites represent new targets of protective microbiota, such as *Deinococcus radiodurans*, which can cooperate to resist chronic RI (Shuryak et al*.* 2017). On the other hand, enteral nutrition can benefit patients with RI to some extent. A randomized controlled trial was conducted by Shao et al*.* (2014) to study the cooperation between microbiota and enteral nutrition in acute RI, the result proved that the tolerance of the microbial immune enteral nutrition group to intestinal symptoms (including abdominal pain, bloating, and diarrhea) was better than the control group (*P* were 0.018, 0.04 and 0.008 after 7 days; *P* were 0.018, 0.015 and 0.002 after 14 days), and the cellular immune parameters were better than the control group (*P* = 0.008, *P* = 0.039, *P* = 0.032). Herein, combining clinical intervention based on microbiota and adjuvant treatments such as enteral nutrition can achieve personal protection for RI.

### Public education

Patients with cancer who cannot avoid radiation should have regular medical examination to understand their condition, as well as adequate treatments and physicians’ professional advice are also required. Those at high risk can take the medication in advance for preventive treatment. On the other hand, for physicians or health professionals, regular intensive learning and testing provide helpful information and aftercare. Patient’s awareness of potential symptoms and early reporting are as important as a physician’s awareness and proactive management of risks and complications (Mitchell et al*.* 2021). Besides, it is also plausible to popularize radiation damage through the internet, television, and newspapers, as well as to offer courses in schools for students. In addition, the government should provide radiation monitoring program services to the public annually.

### Precise technology

Advances and innovations in radiotherapy technology are a critical step toward precision radiation. FLASH radiotherapy is currently regarded as the next breakthrough in the radiation treatment of cancer. Preclinical studies have shown that FLASH radiotherapy can be delivered with very high radiation doses in very short times and substantially can widen the therapeutic window of radiotherapy (Vozenin et al*.* 2022). Besides, microbeam radiation therapy, a novel technique based on synchrotron-generated and spatially fractionated radiotherapy, has been shown to have exceptional healthy tissue-sparing capabilities while maintaining good tumor control.

With the assistance of visualization instruments, such as imaged-guided brachytherapy and computed tomography or magnetic resonance-guided adaptive radiotherapy, it is possible to adjust the irradiated volume to account for changes in organ and/or tumor position, size, or shape over the course of treatment (Hall et al*.* 2022). Additionally, artificial intelligence is currently being introduced into radiation oncology, and machine learning models allow automation and optimization of the workflow. Due to concerns of radiological terrorism, many institutions attempt to replace radionuclide sources with lower energy X-ray sources, which may be more efficient in preclinical models, but the efficacy and adverse effects will be different (Bell et al*.* 2022).

### Policy making

Considering that the majority of populations may experience some form of radiation in the future, the possibility of suffering RI is a substantial public health issue that deserves global policy analysis. Regardless of the potential nuclear disasters, prevention is the most effective strategy. Nuclear power plants should be site in remote areas and radiation protection facilities in medical institutions should be updated continuously. Some countries with experience should offer expert medical and accident planning advice to others and develop a series of extensive guidelines for dealing with accidents (Gale 2017).

The establishment of post-radiation health management centers worldwide is crucial, especially in countries vulnerable to war and those with nuclear weapons. It is reported that the prevalence of patients with RI symptoms does not mitigate even 6–11 years after radiation treatment, indicating the persistence of RI after radiation. Moreover, symptom severity was significantly associated with higher levels of depression. Besides the professional care, the psychological treatment and the comprehensive care during the follow-up phase should also be focused.

### Partnership

Among the above four aspects, the most important is to form a good partnership with each other. As we know, the Fukushima prefecture experienced a triple disaster—the radiation disasters, the Fukushima Daiichi Nuclear Power Plant accident, and the Great East Japan earthquake. Cross-sector collaboration between the local government, private and public medical sectors, and the community has been ongoing for over 10 years in this disaster-affected area to promote reconstruction (Kobashi et al*.* 2022). By working together, organizations can leverage their unique strengths and resources to develop and implement comprehensive prevention and treatment strategies that can help mitigate the impact of RI on individuals and society.

## Conclusion and perspective

The difficulty of diagnosis and no standard therapy of RI remain an intractable problem in clinical. In the context of the ever-popular radiation toxicity, it is crucial to refine understanding of RI, explore novel approaches to effectively manage and mitigate the radiation toxicity in populations. This article reviewed the possible mechanism of RI, and highlighted the microbiota-based treatments for RI. The 5P-Framework will help to improve the understanding and awareness of radiation damage throughout society, providing a comprehensive strategy for managing RI. In conclusion, comprehending the intricate involvement of gut microbiota in the occurrence and management of RI has the potential to advance the field of microbiota medicine, facilitating both the prevention and treatment of RI.
